# Pessimism and Optimism in the Debate on Climate Change: A Critical Analysis

**DOI:** 10.1007/s10806-021-09865-0

**Published:** 2021-07-08

**Authors:** Anders Nordgren

**Affiliations:** Centre for Applied Ethics, Linköping University, 581 83 Linköping, Sweden

**Keywords:** Climate change, Ideology, Mitigation, Optimism, Pessimism, Uncertainty

## Abstract

In the debate on climate change commentators often express pessimistic or optimistic views. We see this mainly in the media and popular literature, but also in various academic fields. The aim of this paper is to investigate different kinds of pessimistic and optimistic views put forward in this debate and suggest explanations of the diversity of views. The paper concludes that pessimism and optimism may concern, for example, climate change as an unmitigated or poorly mitigated process, mitigation of climate change or specific measures of mitigation. These aspects are important to distinguish, because a person can be pessimist concerning climate change as an unmitigated or poorly mitigated process and optimist concerning mitigation of climate change, and be pessimist concerning one specific mitigation measure and optimist concerning another. It is suggested that the diversity of pessimistic and optimistic views is due to the uncertainty of scientific climate models and the influence of evaluative and ideological assumptions.

## Introduction

In the debate on climate change commentators often express pessimistic or optimistic views. We see this mainly in the media and popular literature (for example, Boyd, 2015; Foer, 2019; Franzen, 2019; Gates, 2021; Klein, 2019; Scranton, 2015, 2018; Wallace-Wells, 2017, 2019), but also in various academic fields such as climate science (Lenton et al., 2019; Ripple et al., 2020), economics (Grubb, 2018; McAfee, 2019; Pettifor, 2019; Schröder & Storm, 2018), political science (Røgeberg et al., 2010; Halal & Marien, 2011; Heatley, 2017; Symons, 2019), sociology (Norgaard, 2011), psychology (Clayton, 2018; Lertzman, 2015), philosophy (Brei, 2016; Brooks, 2021; Fiala, 2016; Gardiner, 2006, 2011; McKinnon, 2014), and literature studies (Murphy, 2014).

The aim of this paper is to investigate different kinds of pessimistic and optimistic views put forward in this debate and suggest explanations of the diversity of views. The investigation has no ambition of being all-encompassing but focuses on key examples of different positions and arguments illustrating the diversity. The texts selected for analysis use explicitly the terms ‘optimism’ or ‘pessimism’ or express views that reasonably can be described by these terms. Moreover, the selected texts represent a variety of disciplines. The analysis is critical in the sense that positions and arguments are put into scientific, evaluative and ideological perspective.

In order to carry out the analysis, a few conceptual clarifications (or analytic tools) are needed. First, ‘pessimism’ and ‘optimism’ can be understood in different ways, for example as:

1. a psychological process or state of expecting a negative or positive, respectively, outcome of processes or actions,
2. an argued position of expecting a negative or positive, respectively, outcome of processes or actions.

Second, in the context of climate change, pessimism and optimism may concern different aspects. A person can be a pessimist regarding some aspects and an optimist regarding others. For example, pessimism and optimism may concern:

- climate change as an unmitigated or poorly mitigated process,
- mitigation of climate change,
- specific measures of mitigation of climate change,
- adaptation to climate change, or
- specific measures of adaptation to climate change.

In this paper the main focus will be on argued positions concerning climate change as an unmitigated or poorly mitigated process (2a), mitigation of climate change (2b) and specific measures of mitigation of climate change (2c). However, I will also to a limited extent investigate (2d) and (2e). I will not analyse pessimism/optimism as a psychological process or state (1).

In order to clearly see the relevance of these distinctions, let us have a look at the following quotation from Symons’ book on ecomodernism:

Ecomodernists are often called techno-optimists. The term puzzles me. Most ecomodernists are pessimistic about the likely trajectory of climate change–they think warming above 2°C is now inevitable. Most are pessimistic about the potential for existing technologies to achieve decarbonization … They are pessimistic about proposals for intermittent renewable energy … [E]comodernists are pessimistic about the prospects for international cooperation … So why are ecomodernists called optimists? I think it’s because, despite the real risk that climate change will prove catastrophic, they argue that through the wise use of science a ‘good Anthropocene’ could be possible (Symons, 2019, 25–26).

We see here that ecomodernists are considered pessimists concerning climate change as an unmitigated or poorly mitigated process (2a). Moreover, they are pessimists concerning various specific mitigation measures such as the use of existing technologies, renewable energy and international cooperation (2c). Nevertheless, ecomodernists are optimists concerning certain other mitigation measures (2c) and mitigation in general (2b). Through a “wise” application of science they expect a good future for humankind. So, ecomodernists are pessimists concerning some aspects and optimists concerning others. As we will see below, this holds also for other positions in the debate on climate change.

The various positions on pessimism/optimism concerning climate change are based on certain assumptions about climate change. In my analysis I focus only on positions that accept the following assumptions:

(i) climate change is real,
(ii) climate change is to a substantial extent caused by human action (mainly the use of fossil fuels), and
(iii) climate change is a serious and urgent problem.

Climate optimism based on a denial of the reality of climate change, or on a denial that it is to a substantial extent caused by human action, or on a denial that it is a serious and urgent problem, will not be discussed. Positions accepting (i), (ii) and (iii) may still differ on (iii), that is, on how serious and urgent the problem of climate change is thought to be.

Given the focus on three separate-but related-issues (2a, 2b, 2c), the paper is divided into three main sections. The first section investigates scientific views on the likelihood of disaster due to unmitigated or poorly mitigated climate change. The second section focuses on a few key examples of pessimistic and optimistic views on mitigation and on how these views are related to views on unmitigated or poorly mitigated climate change, mitigation measures and adaptation. The third section digs deeper into the issue of pessimism and optimism concerning mitigation measures and analyses systematically numerous arguments on this issue, including several arguments not investigated in the second section.

## Unmitigated or Poorly Mitigated Climate Change

The first aspect to be investigated is climate change as an unmitigated or poorly mitigated process (2a above). As mentioned, I set aside optimists in this regard, that is, commentators who deny that climate change is real (i) or believe that it is not a serious and urgent problem (iii). The focus is on scientific views and these show varying degrees of pessimism.

### Degrees of Pessimism

The severity and urgency of global warming has been stressed in a recent statement signed by an overwhelming number of scientists:

Scientists have a moral obligation to clearly warn humanity of any catastrophic threat and to “tell it like it is.” … [W]e declare, with more than 11,000 scientist signatories from around the world, clearly and unequivocally that planet Earth is facing a climate emergency (Ripple et al., 2020).

This strong pessimism regarding climate change as an unmitigated or poorly mitigated process (2a above) is also expressed by Lenton and colleagues (Lenton et al., 2019). They argue for a highly increased risk of tipping points such as loss of the West Antarctic ice cap, elimination of the Amazon rainforest and thaw of permafrost in Arctic regions:

In our view, the evidence from tipping points alone suggests that we are in a state of planetary emergency: both the risk and the urgency of the situation are acute … We argue that the intervention time left to prevent tipping could already have shrunk towards zero, whereas the reaction time to achieve zero emissions is 30 years at the best. Hence we might already have lost the control of whether tipping happens. A saving grace is that the rate at which damage accumulates from tipping–and hence the risk posed–could still be under our control to some extent (Lenton et al., 2019).

However, Lenton and colleagues recognize that there are some scientists who believe that global tipping is “highly speculative” with “low probability” (Lenton et al., 2019). This disagreement indicates some differences in degree of pessimism concerning climate change as an unmitigated or poorly mitigated process.

Another example of differences in degree of pessimism can be seen in the debate on one of the climate scenarios presented in the 2014 IPCC report, namely the RCP8.5 scenario. This scenario was one of four scenarios (Representative Concentration Pathways, RCPs) for what might happen with emissions and temperature rise by 2100. RCP8.5 (an RCP with an additional 8.5 W/m^2^ radiative forcing) “projects” a 4–5 °C rise above preindustrial levels with disastrous climate impact (IPCC, 2014, 57–66). The term ‘projection’ should not be confused with ‘prediction.’ While the latter term refers to what is likely to happen, the former only explores what may happen given certain assumptions regarding emissions levels, temperature and climate impact (King et al., 2015, 29–30). In the 2014 IPCC report the RCP8.5 was never intended to predict the future; it was merely a projection. However, it has been used by many commentators as a prediction of what is likely to happen in absence of strong and effective mitigation policies, that is, as a result of “business-as-usual” (Hausfather & Peters, 2020). Hausfather and Peters argue that the business-as-usual interpretation is misleading. RCP8.5 is only a “highly unlikely” worst-case scenario requiring an increase in the use of coal that exceeds some estimates of the accessible coal reserves and an unlikely reversal of the current trend of declining costs of clean energies relative fossil fuels. According to many studies we are rather heading towards 3 °C above preindustrial levels by 2100, which is serious enough (Hausfather & Peters, 2020). To this has been responded that the RCP8.5 scenario could still be likely due to feedbacks and tipping points such as thaw of permafrost in the Arctic releasing vast amounts of methane (cf. Lenton et al., 2019 cited above). Moreover, among the four scenarios RCP8.5 agrees best with the historic cumulative CO2 emissions 2005–2020 and matches best the current and stated climate policies (Schwalm et al., 2020).

So, scientists generally agree that climate change is a serious and urgent problem but show some disagreement on exactly how serious and how urgent. They are to a varying extent pessimistic concerning its trajectory and adverse effects. Examples of adverse effects are extreme weather events, flooding, drought, wildfires, sea-level rise, destruction of ecosystems, extinction of species, sustainability problems for indigenous populations, spreading of diseases, unequal global distribution of harm (low-income countries are likely to suffer more than high-income countries), migration (climate refugees), reduced global food security (for example, famines), and socio-economic tensions (resulting, for example, in social collapse or war) (IPCC, 2014, 64–74). Some of these adverse effects can to some extent already be seen at 1 °C above preindustrial levels. Moreover, they are projected to become worse at 1.5 °C, and even more so at 2.0 °C (IPCC, 2018), which are the goals of the COP21 Paris Agreement (UNFCCC, 2015). With the present momentum global warming is heading towards 3 °C by 2100 (Hausfather & Peters, 2020) or even higher (Schwalm et al., 2020) with further disastrous impact.

### Comments

In the scientific debate we find some differences concerning the trajectory and adverse effects of climate change as an unmitigated or poorly mitigated process. Most scientists seem to be very pessimistic, some somewhat less so. These differences raise several questions. To what extent can science “tell it like it is”, as stated by Ripple and colleagues in the quotation above? Climate change is a serious and urgent problem, but to what extent? Do we have scientific reasons for being more pessimistic rather than less, or the other way around?

An important explanation to the disagreement on the trajectory and impact of climate change is the uncertainty of climate models (Kunreuther et al., 2014). Climate systems are complex, dynamic and non-linear. The models simulating them are therefore characterized by uncertainty. The uncertainty may be due to, for example, uncertainty about initial conditions (observations) and future emissions (Gettelman & Rood, 2016, 228). Uncertainty may concern the climate system response, that is, the relation between emissions and temperature rise. This rise is “likely” in the range of 0.8–2.5 °C per trillion tonnes of carbon emitted as CO2 (IPCC, 2014, 62), which is a very large span. Moreover, uncertainty may concern the impact of a particular temperature rise. In addition to these physical factors, uncertainty is also due to social, economic, technological and political factors. Social systems are also complex, dynamic and non-linear. Any assessment of the future trajectory and impact of climate change must therefore include also interactions of climate systems and social systems (King et al., 2015, 18–20, 31–32).

With this uncertainty in mind, it becomes clear that science cannot in a simple and straightforward way “tell it like it is.” The uncertainty of models simulating climate systems and social systems opens for various more or less pessimistic interpretations of the trajectory and adverse effects of unmitigated or poorly mitigated climate change.

## Mitigation

Although there are some differences among scientists concerning climate change as an unmitigated or poorly mitigated process (2a above), the differences in terms of pessimism and optimism are much more substantial in the multidisciplinary debate on mitigation of climate change (2b). Mitigation pessimists stress that it is too late or almost too late to effectively mitigate climate change. Mitigation optimists, on the other hand, maintain that it is still not too late or only on the verge of being too late. There is a spectrum of views varying from radical mitigation pessimists to radical mitigation optimists. Let me give a few examples (since the debate is multidisciplinary and international, I will clarify the academic background and geographic origins of the commentators).

### Degrees of Pessimism

At the pessimistic extreme of the spectrum we find the view of Jem Bendell, a British scholar in sustainability leadership. He argues:

[T]he evidence before us suggests that we are set for disruptive and uncontrollable levels of climate change, bringing starvation, destruction, migration, disease and war … My conclusion to this situation has been that we need to expand our work on “sustainability” to consider how communities, countries and humanity can adapt to the coming troubles. I have dubbed this the “Deep Adaptation Agenda,” to contrast it with the limited scope of current climate adaptation activities (Bendell, 2018).

Bendell goes as far as to expect an “inevitable near-term social collapse” (Bendell, 2018). The trajectory and impact of climate change will lead to a world where society as we know it will collapse and will do so very soon. With this in mind, we should try to mitigate as much as possible although this is not likely to be successful. It is simply too late. Therefore, we need to prepare for a radical form of adaptation to the new situation. It is not sufficient to focus on physical adaptation in terms of resilience to extreme weather events, flooding and droughts. We also need psychological adaptation, that is, radically changing our way of living. Bendell calls this view “deep adaptation.”

Roy Scranton, an American writer and scholar in English and environmental humanities, holds a similar pessimistic view on mitigation and suggests similarly that we should focus on adaptation rather than mitigation. He maintains that this view is already widely spread:

From the perspective of many policy experts, climate scientists, and national security officials, the concern is not whether global warming exists or how we might prevent it, but how we are going to adapt to life in the hot, volatile world we’ve created (Scranton, 2015, 17).

According to Scranton, adaptation fundamentally means ‘learning to die in the Anthropocene’ (the title of one of his books), that is, learning to psychologically and culturally cope with the new disastrous world resulting from global warming (Scranton, 2015).

Brian Heatley is another mitigation pessimist, but not as radical as Bendell and Scranton. Heatley is a British scholar working at a green think tank. He argues that COP21 in Paris 2015 was not the success in international climate cooperation it was hoped to be:

In reality, despite the hype, Paris punctures this optimism … Too little has been agreed far too late. The real meaning of Paris is that dangerous climate change by 2100 is now all but inevitable, and that after 2100 it will get worse … The main argument of this piece is that the Green movement has to come to terms with this awful fact; we have failed in our mission to prevent damaging climate change. Acceptance of that will have profound consequences for our politics: while we must of course continue to act to prevent further climate change, we must also begin to prepare for the world as it will inevitably become… (Heatley, 2017).

The situation after the Paris Agreement has become even worse. Damaging climate change is now almost inevitable. It is too late to stop global warming at 1.5 °C or at least 2 °C (the goals of the Paris Agreement) and we are heading towards at least 3–4 °C by 2100. While doing as much as possible to mitigate further warming, we need to begin to prepare for a potentially disastrous situation. Adaptation becomes more and more essential. However, Heatley puts equal emphasis on mitigation and adaptation rather than prioritizing adaptation over mitigation, as Bendell and Scranton seem to do. Heatley’s understanding of adaptation is also different. He does not focus on psychological and cultural adaptation but rather on physical adaptation, that is, on aspects such as food security, energy security and national security (Heatley, 2017).

Thom Brooks, an American-British philosopher and legal scholar, is less pessimistic than Heatley. He represents a middle position in between pessimism and optimism concerning mitigation. He describes himself as “a realistic optimist” (Brooks, 2021, 88). However, as I interpret him, his ‘realism’ actually seems to be a kind of pessimism. This is how Brooks summarizes his view:

I have argued that existing ways of thinking about the climate–specifically as a problem in search of a “solution”–is deeply flawed. The climate cannot be stopped from changing and there is no off button, but we can influence how much (or less) it changes. So there is no end-state fixed solution, no happily ever-after. If only we consumed much less or made polluters pay for their pollution, these efforts will not prevent an environmental catastrophe in future … But this inevitability of future catastrophe is not a reason to do nothing or see our efforts as pointless … We need to mitigate more … We need to invest and develop urgently in new adaptive technologies (Brooks, 2021, 82–83).

Three things are important here. First, we see an element of mitigation pessimism. We cannot prevent climate change. A future environmental catastrophe is inevitable. Even if we can influence the degree of disaster to some extent, we cannot solve the problem once and for all. Brooks criticizes what he conceives as the “orthodox” view on sustainability, namely that sustainability is a permanent state of total security. As a more realistic alternative, Brooks suggests the more modest goal of “impermanent sustainability.” Climate change lacks a fixed solution. We must learn to live with climate change and continually readjust our measures (Brooks, 2021, 66–67). Second, there is an element of mitigation optimism. We should not see our efforts as meaningless. We can, at least to some extent, influence how much the climate changes. Third, as Heatley, Brooks puts equal emphasis on mitigation and adaptation. We should do all we can to mitigate climate change and to adapt to its adverse effects. Notice also that Brooks’ focus concerning adaptation is on technologies, as with Heatley, not on psychological or cultural adaptation, as with Bendell and Scranton.

### Degrees of Optimism

Let us turn to the other extreme of the pessimism/optimism spectrum, namely radical mitigation optimism. Here we find the authors of *An Ecomodernist Manifesto*. Accepting a pessimistic view of climate change as an unmitigated or poorly mitigated process, ecomodernists nevertheless expect positive social transformation by means of high-tech innovation. The authors of *An Ecomodernist Manifesto* represent a variety of academic fields, including ecology, physics, economics and philosophy. Most are Americans, but some originate in Australia and one author in India (Asafu-Adjaye et al., 2015). They express their optimism as follows:

We offer this statement in the belief that both human prosperity and an ecologically vibrant planet are not only possible but also inseparable … [W]e embrace an optimistic view toward human capacities and the future (Asafu-Adjaye et al., 2015).

In stark contrast to Scranton’s pessimistic view of the Anthropocene, the authors of *An Ecomodernist Manifesto* have a much brighter outlook:

[K]nowledge and technology, applied with wisdom, might allow for a good, or even great, Anthropocene (Asafu-Adjaye et al., 2015).

Key to successful mitigation of climate change is technology:

Meaningful climate mitigation is fundamentally a technological challenge. By this we mean that even dramatic limits to per capita consumption would be insufficient to achieve significant climate mitigation … In the long run, next-generation solar, advanced nuclear fission, and nuclear fusion represent the most plausible pathways toward the joint goals of climate stabilization and radical decoupling of humans from nature (Asafu-Adjaye et al., 2015).

The goal of “climate stabilization” is linked to the goal of “decoupling of humans from nature,” not to the goal of most environmentalists of harmonizing human society with nature. By decoupling we will preserve the environment and at the same time increase human prosperity. This decoupling is to be achieved by means of technological development and intensification of, for example, urbanization and agriculture (Asafu-Adjaye et al., 2015).

Another kind of ecomodernism is proposed by the Australian scholar in politics and international relations Jonathan Symons (quoted in the Introduction). Symons differs from the authors of *An Ecomodernist Manifesto* mainly by emphasizing the role of the state more than they do in their liberal version. He describes his kind of ecomodernism as guided by the idea of “global social democracy” and stresses solidarity with poor people in low-income countries (Symons, 2019, 167, 182–188, 196–198). State-led mission-oriented high-tech innovation is key to mitigating climate change (Symons, 2019, 111–123). In particular, he lifts the potential of geoengineering (Symons, 2019, 173–182, 188).

A completely different type of optimism we find in David Boyd’s book *The Optimistic Environmentalist* (2015). Boyd, a Canadian environmental lawyer, presents his view as follows:

My goals in writing this book are to provide an antidote to the plague of ecological negativity; to give people a sense of optimism that a greener, cleaner, and happier future is possible; and to inspire to act. A bright future isn’t a 99 to 1 longshot, but rather the odds-on favourite … [M]y conviction that a sustainable and prosperous future lies within our reach is based on a sober and balanced examination of the facts about humanity’s past environmental successes, current trends, and future probabilities (Boyd, 2015, xxiii).

It should be noted that climate change and mitigation of climate change are not central to Boyd’s book and not given priority over other ecological issues such as pollution, biodiversity and living in harmony with nature. However, while recognizing “the potentially catastrophic effects of climate change” caused by fossil fuel use, he indicates a mitigation optimist stance by stressing that “the transition to clean, renewable energy is inevitable and accelerating” (Boyd, 2015, 25–26). This said, it is still clear that Boyd argues for green optimism in general rather than mitigation optimism more specifically. He expects “a future where the human impact on the planet is reduced to sustainable levels and the ravaged ecosystems of today are restored to flourishing levels of diversity and abundance” (Boyd, 2015, xxv). As indicated by the quotation above, his green optimism is based on three types of argument: examples of historical successes such as regulations against species extinction and the 1987 Montreal agreement to protect the ozone layer, examples of current trends such as the renewable energy revolution (solar and wind power) and the increasing popularity of organic food, and examples of likely further developments in, for instance, electric transportation and energy-efficient building (Boyd, 2015).

Another optimistic approach with a more direct focus on mitigation of climate change than Boyd’s and with an emphasis also on economic justice can be seen in a recent book by the British economist Ann Pettifor (2019). In the spirit of Roosevelt’s New Deal in the US in the 1930s, she argues for a UK Green New Deal similar to the US Green New Deal suggested by Alexandria Ocasio-Cortez and Ed Markey (Pettifor, 2019, 7–20). The following quotation clearly illustrates the difference between the more traditional green approach to climate change mitigation (such as Boyd’s) and the Green New Deal:

Environmental advocates tend to focus on individual (‘change your lightbulbs’) or community (‘recycle, reuse, reduce, localise’) action. We have been slow at understanding and promoting the need for radical systemic change across sectors and at a global and national level; that is, change that involves state action … To tackle climate change we need simultaneously to tackle the root cause of growing toxic emissions: a self-regulating, globalised financial system … Once we switch off the ‘tap’ of ‘easy money’ will it be possible to switch off the flow of oil and other fossil fuels (Pettifor, 2019, 8–9).

We see here that Pettifor stresses the role of the state in mitigation of climate change and not merely, as environmentalists (including Boyd) often tend to do, the role of individuals and the community. The fundamental factor behind climate change is the global financial system that upholds the fossil fuel industry. In order to successfully mitigate climate change the state needs to take control over, regulate and reform this financial and monetary system. This is how Pettifor expresses optimism concerning this basic mitigation measure:

There is hope; and it rests not on a utopian vision of humanity, but on our knowledge of human genius, empathy, ingenuity, collaboration, integrity and courage. We know that it is possible to reform the globalised financial system because we have done it before–and in the relatively recent past … To transform the current economic and financial system we must ignore defeatists on both the left and right of the political spectrum… (Pettifor, 2019, xvi–xvii).

It is not too late to mitigate climate change on the basis of such a reformation of the financial system. It can be done, because a similar reformation was done “in the relatively recent past,” namely by Roosevelt’s dismantling of the gold standard in the 1930s as part of the New Deal (Pettifor, 2019, 48–50). The economic goal of the Green New Deal is a ‘steady state’ economy (circular economy) in which mitigation measures are integrated with measures for economic justice (Pettifor, 2019, 66–67). This economic system is mixed in the sense that the state undertakes certain collective activities and the market facilitates exchange of goods and services within the steady state framework (Pettifor, 2019, 99). The Green New Deal is described as a “democratic eco-socialist” project (Pettifor, 2019, 59).

See Table 1 for an overview of the positions presented above.

**Table 1:** Overview of combinations of pessimistic/optimistic views on unmitigated or poorly mitigate climate change, pessimistic/optimistic views on mitigation in general, views on mitigation measures, and views on the significance of adaptation (the commentators are presented in an order corresponding to the order of presentation in the text)

	Unmitigated or poorly mitigated climate change	Mitigation	Mitigation measures	Significance of adaptation
Bendell	Radical pessimism	Radical pessimism	Too late	Prioritized
Scranton	Radical pessimism	Radical pessimism	Too late	Prioritized
Heatley	Pessimism	Pessimism	Almost too late	Emphasized: equal consideration
Brooks	Pessimism	Pessimism/optimism (a middle position)	Some measures may have at least a limited effect	Emphasized: equal consideration
Ecomodernist Manifesto	Pessimism	Radical optimism	High-tech innovation is very promising	Not emphasized
Symons	Pessimism	Optimism	State-led high-tech innovation is promising	Not emphasized
Boyd	Pessimism	Optimism	Several efforts to harmonize with nature are promising	Not emphasized
Pettifor	Pessimism	Optimism	Transformation of the financial system is promising	Not emphasized

### Comments

We have seen that mitigation pessimists support their views by referring to pessimistic interpretations of climate change as an unmitigated or poorly mitigated process. It is too late (Bendell, Scranton) or almost too late (Heatley) for successful mitigation, and we should therefore rather focus on adaptation (Bendell, Scranton) or at least give mitigation and adaptation equal consideration (Heatley). Mitigation optimists admit that climate change as an unmitigated or poorly mitigated process constitute a serious and urgent problem but argue that there is still time to do something about it (*Ecomodernist Manifesto*, Symons, Boyd, Pettifor). They highlight potentially promising mitigation measures, referring to historical examples and examples of current trends. This optimism concerning certain mitigation measures functions as argument for their optimism concerning mitigation as such. Brooks, representing a middle position in between mitigation pessimism and mitigation optimism, criticizes the idea of permanent sustainability. He stresses that this goal will never be attained. A future environmental catastrophe cannot be avoided. Mitigation measures can only be of limited value. Still, they are not pointless. Moreover, adaptation measures are of equal significance.

In proposals like these on mitigation of climate change, political ideologies play a central role. A political ideology not only provides a conceptual and evaluative framework for views on mitigation (and adaptation), by its background assumptions about determinants of social change it may also influence what can be the expected outcome of various mitigation measures. In this way, a political ideology may support pessimistic or optimistic views on specific mitigation measures and thereby indirectly support pessimistic or optimistic views on mitigation as such.

Some mitigation optimists embrace a technology-focused liberal ecomodernism (*Ecomodernist Manifesto*) or a social democratic ecomodernism emphasizing the role of the state in technological development (Symons). Others draw on a traditional green ideology stressing individual responsibility and the development of a community in harmony with nature (Boyd). Others again ground their mitigation proposals on a democratic eco-socialist ideology focusing on the role of the state in transforming the global financial system that supports fossil fuel capitalism (Pettifor). All these different ideologies imply that it is possible to change society in a desired direction and may in this way support mitigation optimism.

Political ideology may also play a role in supporting mitigation pessimism, although this view seems to be based primarily on pessimistic interpretations of climate change as an unmitigated or poorly mitigated process. Mitigation pessimism implies pessimism concerning all potential specific mitigation measures. Some potential measures are considered politically acceptable, although their implementation is deemed to come too late. Other potential measures are viewed with pessimism because they are considered politically misguided (for example, eco-fascist measures). Moreover, the various suggestions concerning the adaptation that is to replace or be considered equally with mitigation seem also influenced by political ideology. While some focus on physical adaptation (Heatley, Brooks), others go deeper and emphasize also psychological and cultural adaptation (Bendell, Scranton). Here evaluative and ideological concerns seem to play a central role.

## Specific Mitigation Measures

We will now dig deeper into the issue of pessimism and optimism concerning specific mitigation measures (2c above). Although I now and then refer to some of the commentators investigated in the previous section, I focus to a large extent on other authors. The reason is that these other authors often present key arguments in a more elaborate way and that several important arguments are not mentioned by the commentators analysed in that section.

I focus on three types of mitigation measures: economic and technological measures, political measures and measures undertaken by individuals.

### Economic and Technological Measures

Three issues are of particular importance when it comes to economic and technological mitigation measures: the power of the fossil fuel industry, growth and emissions, and technology and energy. They are all interrelated.

#### The Power of the Fossil Fuel Industry

Major commercial and state-owned fossil fuel and cement companies play an extremely central role in anthropogenic climate change. 63% of global industrial carbon dioxide and methane emissions between 1750 and 2010 can be traced to 90 of these companies (Heede, 2013). Pessimists emphasize that “powerful vested interests in the fossil-fuel industry are resisting change” and this makes the success of a transition to renewable energy less probable, even if some fossil fuel companies do undertake some measures in this direction (Schröder & Storm, 2018).

To this kind of argument optimists may respond in different ways. One option is to stress that this picture of the fossil fuel industry is too dark. Eventually these companies will recognize that they can make profits by investing in an energy transition and thereby create new job opportunities as well as meet the demands of consumers who prefer zero-carbon products. Moreover, optimists may argue that capitalism has a dynamic of its own leading to an energy transition. Capitalism together with a ‘responsive government’ is the solution to the problem. This is stressed, for example, by McAfee (2019, 183–189, 265–268). An opposite approach is suggested, for example, by Pettifor and Klein. They argue that capitalism is the problem, not the solution. What is needed is a democratic eco-socialist transformation of society (Pettifor, 2019, 59; Klein, 2019, 250–252).

#### Growth and Emissions

The role of economic growth (measured by Gross Domestic Product, GDP) in mitigation of climate change is heavily contested. Some commentators maintain that economic growth is the basic cause of global warming. Others argue that it is possible to decouple growth and resource use as well as growth and greenhouse gas emissions. Such decoupling makes continued growth possible, while at the same time resource use and emissions can be reduced.

Pessimists concerning the mitigation option of decoupling maintain that historical evidence suggests that permanent and absolute (not merely relative) decoupling of growth and resource use at a global scale is not possible, although it may occur in high-income countries under very optimistic conditions and in the shorter term. Moreover, they argue that while permanent absolute decoupling of growth and emissions is technically possible nationally as well as globally in line with the 2015 Paris Agreement of attaining the goals of 1.5 °C or at least 2 °C, it is highly improbable even under very optimistic conditions and in the long run (Hickel & Kallis, 2019; see also Schröder & Storm, 2018; Parrique et al., 2019). This makes reduced use of resources and reduced emissions necessary. There are limits to growth (Meadows et al., 1972). We need to recognize these limits and find ways of living within planetary boundaries. Reduced use of resources and reduced emissions will save the planet and mitigate climate change (O’Neill et al., 2018). In line with this Pettifor argues for a Green New Deal aiming at a steady state economy (Pettifor, 2019, 66–67).

Optimists concerning decoupling criticize this view of growth. They think it underestimates the potential of technological innovation and argue that decoupling of growth and resource use as well as growth and emissions is possible (Grubb, 2018; McAfee, 2019, 237–238, 243–246, 261). This ‘green growth’ position is adopted, for example, by the OECD (2011) and the World Bank (2012). Optimists highlight examples such as the strong market growth for solar power in Germany, the UK electricity transition away from coal, and the growth of sales of electric vehicles in California. Grubb stresses that the efforts need not be global to begin with. They start from local niche markets (Grubb, 2018).

#### Technology and Energy

Technology and energy are at the centre of the pessimism/optimism debate. Tech-pessimists stress that existing low-carbon or zero carbon technologies have not yet achieved much in terms of mitigation. And even if it is possible that technological breakthroughs may have an impact in the future, this hardly holds true in the short run. Technological development is simply not rapid enough to meet the climate emergency we already face (Hickel & Kallis, 2019).

Tech-optimists, on the other hand, point out that historically humankind has always come up with new technological solutions to serious problems. They emphasize that technological development often is non-linear. New technology may completely change the agenda (Grubb, 2018).

However, optimists may have very diverging views on which technologies and energy sources may resolve the mitigation problem. Many optimists stress that several green technologies already exist and are likely to be further developed, exemplifying with electric vehicles and energy-efficient buildings (Boyd, 2015, 159–185). Some also argue for not yet developed technologies such as geoengineering, for example, spraying particles into the stratosphere that reflect solar radiation back into space. They view this as a kind of plan B shouldn’t other mitigation measures be sufficient (Symons, 2019, 169–182). Regarding energy much optimism is linked to already existing renewable energy sources such as hydroelectric power, solar power and wind power (Grubb, 2018). Many also highlight technologies under development such as technologies for capturing and storing carbon dioxide with the goal of achieving negative emissions (Symons, 2019, 91, 106). Some tech-optimists stress the importance of continued use of nuclear power and development of 4th generation fission reactors. They may also suggest further research on nuclear fusion (Asafu-Adjaye et al., 2015). However, proponents of nuclear power often admit that it meets strong resistance in many countries and among most environmental organizations, making this option less feasible (McAfee, 2019, 250–252).

#### Comments

Commentators commonly agree that specific mitigation measures should be science-based, that is, take seriously the best available scientific evidence, including evidence from both natural and social sciences. However, regarding all three interrelated issues—the power of the fossil fuel industry, economic growth, and technology—it is obvious that ideological assumptions influence whether commentators come out as pessimists or optimists. The scientific evidence is not sufficiently conclusive, and this opens for different interpretations. Pessimism and optimism concerning specific mitigation measures are at least partly influenced by ideological assumptions.

Even if many commentators agree that the vested interests of the fossil fuel industry to continue with fossil fuel use need to be stopped in order to mitigate climate change, there are different views on how to achieve this goal. And these different approaches are influenced by ideological assumptions. Some optimists believe that capitalism’s own dynamics with governmental support will lead to an energy transition (McAfee, 2019), while some pessimists concerning this option maintain that a more radical transformation of society is necessary (Klein, 2019; Pettifor, 2019).

The situation is similar regarding the role of economic growth. Is growth the fundamental cause of climate change or part of the solution? This is not just a matter of science. Here the influence of ideological assumptions becomes particularly obvious. Some argue that there are limits to growth and that we need a steady state economy in order to mitigate climate change. They are pessimistic concerning (absolute) decoupling of growth from resource use and emissions. This pessimism is sometimes influenced by some version of democratic eco-socialist ideology (Klein, 2019; Pettifor, 2019). Others argue that it is possible to decouple growth from resource use and emissions. They may be influenced by a green pro-capitalist ideology (McAfee, 2019) or by liberal (Asafu-Adjaye et al., 2015) or social democratic ecomodernism (Symons, 2019).

Many commentators look for a technological solution to the problem of climate change. They differ, however, on which kind of technology to develop. Supporters of traditional green ideology are optimistic concerning renewable energy and energy-efficient technology (Boyd, 2015). Advocates of liberal or social democratic ecomodernism are pessimistic concerning this option and argue that it is insufficient. They emphasize that we need nuclear power (Asafu-Adjaye et al., 2015) or geoengineering (Symons, 2019) for successful mitigation of climate change. So, also when it comes to pessimism and optimism concerning specific technologies ideological assumptions play a crucial role.

### Political Measures

In this section I focus on two issues: national politics and international cooperation.

#### National Politics

Pessimists regarding national politics may point out many different obstacles for successful national mitigation policies. First, politicians may simply disagree on the severity of the climate change problem and on how to prioritize various mitigation options. Second, they may not dare to be radical enough (for example, introducing high carbon taxes), because of fear of not being reelected. The risk is therefore that the measures become merely virtue signalling politics. Politicians only pretend doing something. Third, not until the situation is becoming extremely serious politicians may start to do something and then it may be too late. Mitigation measures will then be very costly and perhaps too radical to be acceptable among the general public. Fourth, successful mitigation requires international cooperation and it is tempting for politicians to be free-riders not truly committed to far-reaching international agreements (see further below). Fifth, some influential countries exhibit worrying tendencies of not living up to the commitments of the 2015 Paris Agreement, and thus making it extremely difficult to attain the goals of 1.5 °C or at least 2 °C (see below).

Optimists regarding national politics, on the other hand, may stress the economic advantages of climate change mitigation for individual countries. As Stern points out: “… most of what is necessary for emissions reductions over the next two decades is in the self-interest of the individual nations” (Stern, 2015, 85; for an analysis of this kind of argument, see Nordgren, 2016). Green technology and an energy transition away from carbon may lead to more energy-efficient industrial production (Green, 2015) and have co-benefits in terms of improved health and reduced air pollution (Karlsson et al., 2020). Moreover, it may be less costly for nations to prevent adverse effects of climate change by early reduction of emissions than by initiating adaptation measures at a later stage (given that the mitigation measures actually lead to a reduction). Another approach to national politics is suggested by the proposal for a Green New Deal. Pettifor stresses the necessity of the state taking control over the financial and monetary system in order to mitigate climate change. She also expresses optimism regarding the success of such an endeavour (Pettifor, 2019, xvi–xvii, 8–9).

In addition to economic advantages, optimists may also stress the value of mitigation for national security reasons. Mitigation is a matter of self-defence. Many adverse effects of climate change may threaten national security. Extreme weather events, famine, climate migration and social tensions may undermine social stability and provide a ground for terrorism and war (Hagel, 2015). Mitigation may reduce these risks (for an analysis of this kind of argument, see Nordgren, 2016).

So, according to optimists—for economic and national security reasons—less short-sighted and more open-minded politicians may introduce mitigation measures that make a difference. And even if the political situation looks rather murky in some powerful countries, the situation may quickly change to the better. Political systems are dynamic and non-linear and may reach critical points (tipping points) at which politicians start doing what is rational and beneficent after the next election and in the long term.

#### International Cooperation

This brings us to the next issue: Is it in national self-interest to cooperate internationally in order to mitigate climate change? Are international agreements on emission reductions likely and will they be sufficiently efficient? A fundamental problem for international cooperation is that climate change is a global commons problem. Pessimists regarding international cooperation commonly use the more pessimistic designation ‘tragedy of the commons.’ The need for global solutions on climate issues is counteracted by narrow-minded and short-sighted national interests. Free-riding is too convenient (Gardiner, 2006). In addition to these theoretical aspects, pessimists may also stress the sad history of unsuccessful international climate collaborations. A decade ago, Røgeberg and colleagues concluded that the Kyoto Protocol was “a largely ineffective treaty” and that “the history of the Kyoto Protocol confirms the case for pessimism more than it does the contrary position” (Røgeberg et al., 2010). More recently, Schröder and Storm commenting on the 2015 Paris Agreement point out that “[t]he early optimism about the Paris COP21 is giving way to a widespread pessimism that the COP21 will not be working soon enough” (Schröder & Storm, 2018). The global emissions have not been reduced, but rather increased, despite all climate conferences. Some important countries have not been willing to commit themselves sufficiently (Heatley, 2017; Schröder & Storm, 2018).

Optimists may respond in at least four ways. First, the tragedy of the commons can be avoided, as indicated by the economic and national security arguments mentioned above. To a large extent it is in national self-interest to mitigate climate change (Stern, 2015). Second, McAfee argues that the problem can be handled by “the four horsemen of the optimist: capitalism, tech progress, responsive government, and public awareness” (McAfee, 2019, 265; see also 183–189, 265–268). Third, Posner and colleagues maintain that international collaboration could be more successful if it is decoupled from issues of justice. A fundamental problem with recent attempts of international collaboration is that collaboration has been linked to issues of corrective justice (for example, the polluter-pays principle) or distributive justice (for example, the ability-to-pay principle). This linkage has made some rich countries reluctant to commit. A decoupling could make them more willing. Based on a cost–benefit analysis, they may conclude that they are better off with collaboration than not. They may collaborate, for example, out of self-interest or a wish to help countries in need (see Posner & Sunstein, 2008; Posner & Weisbach, 2010). Fourth, history shows that international collaboration on environmental issues has sometimes been successful. A key example is the Montreal Protocol 1987 concerning protection of the ozone layer (Boyd, 2015, 96–99).

#### Comments

It is rather uncontroversial to maintain that mitigation of climate change should and must include political measures. However, it is highly contested to what extent politics should govern mitigation and how much should be left to the market, what the specific political measures should be (for example, cap-and-trade, carbon taxes or regulation of the global financial system) and at what level political decisions should be made (national or international). Views on extent, content, and level are all influenced by ideological assumptions. Moreover, pessimism/optimism concerning what national politics and international cooperation can achieve and which specific political measures can be successfully implemented is also influenced by ideology. As illustrated, there are many diverging views on this.

In practice the political results are so far very limited. Nationally, most politicians announce a willingness to do something and many countries have formulated climate goals, but not much happens. Internationally, there has been a long series of climate conferences, but these have not yet resulted in much, either. It seems fair to conclude that so far not much has been achieved nationally and internationally. The global greenhouse gas emissions are still increasing towards disastrous levels (Ripple et al., 2020; Schwalm et al., 2020; Hausfather & Peters, 2020; see the section “Unmitigated or Poorly Mitigated Climate Change”).

However, it is still an open question whether national politics and international cooperation can be at least partially successful. And even if national politics and international cooperation fail, some hope may still lie with non-linear technological development and non-linear political change. Moreover, a gleam of light is indicated by the recent covid-19 pandemic. This pandemic has shown that politicians may be willing to allocate substantial resources in a severe crisis (Andrijevic et al., 2020). On the other hand, there are big differences between the covid-19 crisis and the climate crisis. While the covid-19 pandemic spread very rapidly and had immediate effects, climate change is slowly evolving with its most severe effects lying in the future. Regarding climate change, it is probably tempting for politicians to stay content with merely virtue signalling politics.

### Measures by Individuals

Not only companies, nations, and global organizations may try to mitigate climate change. Individuals may also undertake mitigation measures, for example making fewer trips by car, making fewer journeys by air, eating less meat and having fewer children (cf. Foer, 2019, 98). In investigating pessimism/optimism concerning mitigation measures to be undertaken by individuals, my focus will be on two issues. The first is psychology, the second activism.

#### Psychology

The tragedy of the commons may occur not only at the national level, as mentioned above, but also at the individual level. Pessimists concerning mitigation measures to be undertaken by individuals may stress the problem of free-riding also at this level. Individuals may recognize that from a collective point of view they should try to mitigate but still refrain from doing so in practice for several reasons. It is too inconvenient or costly. It is difficult to change everyday habits. People feel despair because they believe that it is too late to do anything, that individual action is too insignificant to make any real difference, or that their own ability to make a difference is too limited (Gardiner, 2006). Moreover, many people live their daily lives in a kind of denial of climate change (Norgaard, 2011).

Optimists regarding mitigation measures by individuals may criticize this dark view of human psychology. They may stress that mental and moral resources are available that make individuals prepared to undertake mitigation measures also at some personal cost. People may act out of altruism or a well-understood self-interest. They may act for moral reasons (autonomous obligation) or because social norms prescribe a more climate-friendly way of life (heteronomous obligation) (Skjerve & Lavik, 2019). McKinnon argues that it is flawed to exclude the possibility of individuals to make a difference or to have the ability to make a difference (McKinnon, 2014). Moreover, hope opens “a space for agency between the impossible and the fantastical; without it, the small window in time remaining for us to tackle climate change is already closed” (McKinnon, 2014). Hope can make us act. It is a strong motivational factor and may even be considered a virtue (Kretz, 2016; McKinnon, 2014).

#### Activism

This brings us to climate activism, which is one way for individuals to try to mitigate climate change (Klein, 2019, 1–23). Regarding climate activism among youth, O’Brian and colleagues have differentiated three types. Activists of the first type work within existing structures to influence policies, for example within political parties or NGOs. The second type of activism involves working against power structures to change policies, for example by campaigns, boycotts or disruption of international summits. The third type creates alternative social systems characterized by downscaling of production and consumption (O’Brian et al., 2018). The question is: can climate activists influence politicians and companies to a sufficient degree to make a real difference?

Pessimists may stress the potential weaknesses of each of these three different types of climate activism. Activists who work within existing structures run the risk of merely becoming part of the establishment and have no real influence. Those who work against power structures by boycotts and disruption of meetings will be largely ignored by those with real power in society. The actions will be of no lasting impact on the climate. The most radical activists who try to create alternative social systems include only very few people and become only isolated islands of alternative living without impact on society at large and with no effect on global mitigation of climate change.

Optimists, on the other hand, may point at well-known historical examples such as the British movement for abolition of slavery, Gandhi’s movement against British colonialism in India, women’s rights movement, and the workers’ movement in the nineteenth and twentieth centuries. These activist movements were successful in many respects and show that people can come together, create awareness, and initiate action with snowball effects that change society. This indicates that also climate activism might be successful.

#### Comments

Political ideologies put varying emphasis on the significance of individual action in mitigation of climate change. It is obvious that liberal ecomodernists like the authors of *An Ecomodernist Manifesto* (Asafu-Adjaye et al., 2015) as well as social democratic ecomodernists like Symons (2019) stress high-tech innovation much stronger than individual action. It is also clear that democratic eco-socialists like Pettifor emphasize transformation of the financial system rather than measures by individuals (Pettifor, 2019). In contrast, traditional green ideology has often emphasized the responsibility of individuals as consumers and citizens. By changing their consumption patterns, individuals may influence which products are offered on the market and thereby the greenhouse gas emissions. By exercising their democratic rights to vote, individuals may influence politics in a climate-friendly direction (Boyd, 2015, 198). Basically, proponents of these ideologies are all positive to mitigation efforts by individuals (even if they may not accept all kinds of efforts). However, there are ideology-dependent differences in emphasis and behind these differences in emphasis there are ideology-dependent differences in how pessimistic or optimistic they are concerning the significance of individual action.

## Conclusions

In this paper I have investigated a great variety of pessimistic and optimistic views expressed in the debate on climate change. I have shown that it is problematic to speak about this pessimism and optimism in general terms. Pessimism and optimism may concern different aspects of climate change, for example climate change as an unmitigated or poorly mitigated process, mitigation of climate change or specific measures of mitigation. These aspects are vital to distinguish, because a person can be pessimist concerning climate change as an unmitigated or poorly mitigated process and optimist concerning mitigation of climate change, and be pessimist concerning one specific mitigation measure and optimist concerning another.

Moreover, I have suggested two major explanations of the diversity of pessimistic and optimistic views. The first is the uncertainty of scientific climate models. Climate systems are dynamic and non-linear, and therefore climate models are extremely sensitive to initial conditions (observations) and future emissions. Uncertainty may concern the relation between emissions and temperature rise, and the impact of a particular temperature rise. Further uncertainty is added by the interaction of climate systems and social systems. This uncertainty opens for a variety of interpretations, for example, more or less pessimistic views concerning climate change as an unmitigated or poorly mitigated process.

A second explanatory factor is the influence of evaluative and ideological assumptions. Political ideologies provide conceptual and evaluative frameworks for mitigation proposals. However, by their background assumptions about determinants of social change they may also influence what can be the expected outcome of various mitigation measures. In this way, political ideologies open for a variety of pessimistic and optimistic views on specific mitigation measures and thereby indirectly for a variety of pessimistic and optimistic views on mitigation as such.

## References

[CR1] Andrijevic M, Schleussner C-F, Gidden MJ, McCollum DL, Rogelj J (2020). COVID-19 recovery funds dwarf clean energy investment needs. Science.

[CR2] Asafu-Adjaye, J., Blomquist, L., Brand, S., Brook, B. W., DeFries, R., Ellis, E., Foreman, C., Keith, D., Lewis, M., Lynas. M., & Nordhaus, T. (2015). *An ecomodernist manifesto*. Retrieved May 21, 2021 from www.ecomodernism.org/manifesto.

[CR3] Bendell, J. (2018). *Deep adaptation: A map for navigating climate tragedy*. IFLAS Occasional Paper 2. Retrieved May 21, 2021 from www.lifeworth.com/deepadaptation.pdf.

[CR4] Boyd DR (2015). The optimistic environmentalist: Progressing towards a greener future.

[CR5] Brei AT, Brei AT (2016). Hope and pressure. Ecology, ethics and hope.

[CR6] Brooks T (2021). Climate change ethics for an endangered world.

[CR7] Clayton S (2018). Mental health risk and resilience among climate scientists. Nature Climate Change.

[CR8] Fiala A, Brei AT (2016). Playing a requiem on the titanic: The virtue of hope in the age of ecological calamity. Ecology, ethics and hope.

[CR9] Foer JS (2019). We are the weather: Saving the planet begins at breakfast.

[CR10] Franzen, J. (2019). What if we stopped pretending? The climate apocalypse is coming. To prepare for it, we need to admit that we can’t prevent it. *The New Yorker*, September 8, 2019.

[CR11] Gardiner SM (2006). A perfect moral storm: Climate change, intergenerational ethics and the problem of moral corruption. Environmental Values.

[CR12] Gardiner SM (2011). A perfect moral storm: The ethical tragedy of climate change.

[CR13] Gates B (2021). How to avoid a climate disaster: The solutions we have and the breakthroughs we need.

[CR14] Gettelman A, Rood RB (2016). Demystifying climate models: A users guide to earth system models.

[CR15] Green, F. (2015). *Nationally self-interested climate change mitigation: A unified conceptual framework*. Centre for Climate Change Economics and Policy Working Paper No. 224. Grantham Research Institute on Climate Change and the Environment Working Paper No. 199. Retrieved May 21, 2021 from www.lse.ac.uk/GranthamInstitute.

[CR16] Grubb, M. (2018). *Conditional optimism: Economic perspectives on deep carbonization*. Institute for New Economic Thinking. Retrieved May 21, 2021 from www.ineteconomics.com.

[CR17] Hagel, C. (2015). Climate change is a national security problem. *Time*, December 1, 2015. Retrieved May 21, 2021 from http://time.com/4130796/paris-climate-conference-chuck-hagel/.

[CR18] Halal W, Marien M (2011). Global megacrisis: A survey of four scenarios on a pessimism-optimism axis. Journal of Futures Studies.

[CR19] Hausfather Z, Peters GP (2020). Emissions—The ‘business as usual’ story is misleading. Nature.

[CR20] Heatley B (2017). Paris: Optimism, pessimism and realism. Global Discourse.

[CR21] Heede R (2013). Tracing anthropogenic carbon dioxide and methane emissions to fossil fuel and cement producers, 1854–2010. Climatic Change.

[CR23] Hickel J, Kallis G (2019). Is green growth possible?. New Political Economy.

[CR24] IPCC. (2014). In Core Writing Team, R. K. Pachauri, & L. A. Meyer (Eds.), *Climate change 2014: Synthesis report*. *Contribution of working groups I, II and III to the fifth assessment report of the intergovernmental panel on climate change.* Retrieved May 21, 2021 from www.ipcc.ch.

[CR25] IPCC. (2018). In P. Z. Masson-Delmotte, H. O. Pörtner, D. Roberts, J. Skea, P. R. Shukla, A. Pirani, W. Moufouma-Okia, C. Péan, R. Pidcock, S. Connors, J. B. Matthews (Eds.), *Global warming of 1.5°C. An IPCC special report on the impacts of global warming of 1.5°C above pre-industrial levels and related global greenhouse gas emission pathways, in the context of strengthening the global response to the threat of climate change, sustainable development, and efforts to eradicate poverty.* Retrieved May 21, 2021 from www.ipcc.ch.

[CR26] Karlsson M, Alfredsson E, Westling N (2020). Climate policy co-benefits: A review. Climate Policy.

[CR27] King D, Schrag D, Dadi Z, Ye Q, Ghosh A (2015). Climate change: A risk assessment.

[CR28] Klein N (2019). On fire: The burning case for a green new deal.

[CR29] Kretz L, Brei AT (2016). Singing hope’s praises: A defense of the virtue of hope for environmental action. Ecology, ethics and hope.

[CR30] Kunreuther, H., Gupta, S., Bosetti, V., Cooke, R., Dutt, V., Ha-Duong, M., Held, H., Llanes-Regueiro, J., Patt, A., Shittu, E. & Weber, E. (2014). Integrated risk and uncertainty assessment of climate change response policies. In O. Edenhofer, R. Pichs-Madruga, Y. Sokona, E. Farahani, S. Kadner, K. Seyboth, A. Adler, I. Baum, S. Brunner, P. Eickemeier, B. Kriemann, J. Savolainen, S. Schlömer, C. von Stechow, T. Zwickel & J. C. Minx (Eds.), *Climate change 2014: Mitigation of climate change. Contribution of working group III to the fifth assessment report of the intergovernmental panel on climate change* (pp. 151–205). Retrieved May 21, 2021 from www.ipcc.ch.

[CR31] Lenton TM, Rockström J, Gaffney O, Rahmstorf S, Richardson K, Steffen W, Schellnhuber HJ (2019). Climate tipping points: Too risky to bet against. Nature.

[CR32] Lertzman R (2015). Environmental melancholia: Psychoanalytic dimensions of engagement.

[CR33] McAfee A (2019). More from less.

[CR34] McKinnon C (2014). Climate change: Against despair. Ethics and the Environment.

[CR35] Meadows DH, Meadows DI, Randers J, Behrens WW (1972). The limits to growth: A report to the club of Rome.

[CR36] Murphy PD (2014). Pessimism, optimism, human inertia, and anthropogenic climate change. Interdisciplinary Studies in Literature and Environment.

[CR37] Nordgren A (2016). Climate change and national self-interest. Journal of Agricultural and Environmental Ethics.

[CR38] Norgaard KM (2011). Living in denial: Climate change, emotions, and everyday life.

[CR39] O’Brian K, Selboe E, Hayward BM (2018). Exploring youth activism on climate change: Dutiful, disruptive, and dangerous dissent. Ecology and Society.

[CR40] O’Neill DW, Fanning AL, Lamb WF, Steinberger JK (2018). A good life for all within planetary boundaries. Nature Sustainability.

[CR41] Organisation for Economic Cooperation and Development (OECD) (2011). Towards green growth.

[CR42] Parrique, T., Barth, J., Briens, F., Kerschner, C., Kraus-Polk, A., Kuokkanen, A., & Spangenberg, J. H. (2019). *Decoupling Debunked: Evidence and arguments against green growth as a sole strategy for sustainability*. European Environmental Bureau. Retrieved May 21, 2021 from www.eeb.org.

[CR43] Pettifor A (2019). The case for the green new deal.

[CR44] Posner EA, Sunstein CR (2008). Climate change justice. The Georgetown Law Journal.

[CR45] Posner EA, Weisbach D (2010). Climate change justice.

[CR46] Ripple WJ, Wolf C, Newsome TM, Barnard P, Moomaw WR (2020). World scientists’ warning of a climate emergency. BioScience.

[CR47] Røgeberg O, Andresen S, Holtsmark B (2010). International climate treaties: The case for pessimism. Climate Law.

[CR48] Schröder, E., & Storm, S. (2018). *Why “Green Growth” is an illusion*. Institute for New Economic Thinking. Retrieved May 21, 2021 from www.ineteconomics.com.

[CR49] Schwalm CR, Glendon S, Duffy PD (2020). RCP8.5 tracks cumulative CO2 emissions. Proceedings of the National Academy of Sciences of the United States of America.

[CR50] Scranton R (2015). Learning to die in the anthropocene: Reflections on the end of a civilization.

[CR51] Scranton R (2018). We’re doomed. Now what? Essays on war and climate change.

[CR52] Skjerve K, Lavik T (2019). Don't join the joyride: Individual responsibility for large-scale problems. Etikk i praksis. Nordic Journal of Applied Ethics.

[CR53] Stern N (2015). Why are we waiting? The logic, urgency, and promise of tackling climate change.

[CR54] Symons J (2019). Ecomodernism: Technology, politics and the climate crisis.

[CR55] United Nations Framework Convention on Climate Change (UNFCCC). (2015). *Paris agreement*. Retrieved May 21, 2021 from https://unfccc.int/process-and-meetings/the-paris-agreement/the-paris-agreement.

[CR57] Wallace-Wells, D. (2017). The uninhabitable earth: Famine, economic collapse, a sun that cooks us: What climate change could wreak—sooner than you think. *New York Magazine* July 10, 2017.

[CR58] Wallace-Wells D (2019). The uninhabitable earth: Life after warming.

[CR59] World Bank (2012). Inclusive green growth: The Pathway to sustainable development.

